# Deep learning pneumoconiosis staging and diagnosis system based on multi-stage joint approach

**DOI:** 10.1186/s12880-024-01337-x

**Published:** 2024-07-02

**Authors:** Chang Liu, Yeqi Fang, YuHuan Xie, Hao Zheng, Xin Li, Dongsheng Wu, Tao Zhang

**Affiliations:** 1 West China School of Public Health and West China Fourth Hospital, Sichuan University, Chengdu, 610041 PR China; 2 West China-PUMC C.C. Chen Institute of Health, Sichuan University, Chengdu, PR China; 3 College of Physics, Sichuan University, Chengdu, 610041 PR China; 4 College of Computer Science, Sichuan University, Chengdu, 610041 PR China

**Keywords:** Pneumoconiosis diagnosis, X-rays, Deep learning, Efficient-net, Res-Net34

## Abstract

**Background:**

Pneumoconiosis has a significant impact on the quality of patient survival due to its difficult staging diagnosis and poor prognosis. This study aimed to develop a computer-aided diagnostic system for the screening and staging of pneumoconiosis based on a multi-stage joint deep learning approach using X-ray chest radiographs of pneumoconiosis patients.

**Methods:**

In this study, a total of 498 medical chest radiographs were obtained from the Department of Radiology of West China Fourth Hospital. The dataset was randomly divided into a training set and a test set at a ratio of 4:1. Following histogram equalization for image enhancement, the images were segmented using the U-Net model, and staging was predicted using a convolutional neural network classification model. We first used Efficient-Net for multi-classification staging diagnosis, but the results showed that stage I/II of pneumoconiosis was difficult to diagnose. Therefore, based on clinical practice we continued to improve the model by using the Res-Net 34 Multi-stage joint method.

**Results:**

Of the 498 cases collected, the classification model using the Efficient-Net achieved an accuracy of 83% with a Quadratic Weighted Kappa (QWK) score of 0.889. The classification model using the multi-stage joint approach of Res-Net 34 achieved an accuracy of 89% with an area under the curve (AUC) of 0.98 and a high QWK score of 0.94.

**Conclusions:**

In this study, the diagnostic accuracy of pneumoconiosis staging was significantly improved by an innovative combined multi-stage approach, which provided a reference for clinical application and pneumoconiosis screening.

**Supplementary Information:**

The online version contains supplementary material available at 10.1186/s12880-024-01337-x.

## Introduction

Pneumoconiosis is a category of occupational diseases with the widest impact and the most serious harm. It is a systemic disease caused by fibrosis of lung tissue due to long-term inhalation of productive dust in the production environment. According to the Global Burden of Disease Study [1, 2], there are still much pneumoconiosis, numbering up to 527,500 cases, with more than 60,000 new cases reported globally in 2017, and the mortality rate of pneumoconiosis has stayed at an elevated level in recent years [1, 3]. The 2018 National Occupational Disease Report, released by the National Health Commission, indicated that the number of individuals diagnosed with occupational pneumoconiosis across the country from 2012 to 2018 reached 170,569, representing over 90% of all occupational diseases. The progression of pneumoconiosis is protracted and undetectable, and the established clinical treatments for pneumoconiosis are limited. The only life-saving treatment option for end-stage pneumoconiosis is lung transplantation. Therefore, regular screening of potential risk groups and early detection of the disease is crucial for the early intervention of pneumoconiosis and the improvement of patients’ quality of life.

According to China’s current Diagnosis of Occupational Pneumoconiosis GBZ 70-2015 [4], the core of pneumoconiosis diagnosis is to correctly interpret the X-ray chest radiographs, and make use of the density of small opacities, the distribution of lung areas and other indexes to carry out an appropriate staging of pneumoconiosis patients. At present, the diagnosis and initial screening of pneumoconiosis is done by manual reading of chest radiographs, which is affected by various subjective or objective reasons such as doctors’ experience and level, resulting in misdiagnosis and omission of diagnosis from time to time [5]. The inferior quality of chest radiographs, doctors’ inability to recognize the disease, and the different latent periods of pneumoconiosis have led to serious limitations in the early diagnosis of pneumoconiosis. Early detection of pneumoconiosis and its interventions still need an economical, timely, accurate, and efficient screening solution.

With the continuous deep research of artificial intelligence(AI), medical image-assisted diagnostic testing technology has been gradually applied to medical clinics, to improve the diagnostic stability and accuracy of doctors, there is an urgent need for computer-assisted diagnosis technology Computer-aided diagnosis (CAD) and deep learning technology Deep Learning (DL) on chest radiographs to quickly and accurately CAD and DL are urgently needed for rapid and accurate screening of chest radiographs to assist in diagnosing pneumoconiosis. Since 2010, with the rise of CAD, several researchers have proposed several image analysis methods for diagnosing pneumoconiosis based on the texture features of chest radiographs [6–9] to help radiologists reduce their workloads and improve diagnostic efficiency. However, all these methods require a certain degree of subjective “manual” work by physicians to accomplish tasks such as feature definition, which is challenging for the complex task of pneumoconiosis staging diagnosis. Deep learning techniques have had remarkable success in medical imaging tasks [10]. Several studies [11–13] have demonstrated the feasibility of using AI to diagnose lung abnormalities such as lung nodules, tuberculosis, cystic fibrosis, etc. Several studies have also conducted extensive research on the use of AI in pneumoconiosis diagnosis, including classification [7, 14, 15], detection [16], etc. However, due to the limited number of well-labeled pneumoconiosis chest radiographs for training AI models, the complexity of pneumoconiosis disease itself, and the uneven distribution of staging, some studies only achieve binary classification [17] or single-stage multi-classification [18].For binary classification, it only provides simplistic outcomes, labeling images as either normal or abnormal, without the ability to discern the severity of the condition. This limitation heightens the risk of overlooking or misjudging the extent of the disease. For single-stage multi-classification, this method is susceptible to imbalance issues and lacks robustness in modeling.so there is a lack of AI-based diagnostic tools for pneumoconiosis staging.

To this end, this study leveraged a deep learning-based U-Net model to extract lung fields from pneumoconiosis chest radiographs and employs neural networks to learn image features, enabling precise classification of pneumoconiosis stages. By comparing the proposed multi-stage joint approach model with traditional single neural network models, this research aimed to advance the application of deep learning in pneumoconiosis diagnosis and screening, offering a more accurate and efficient solution.

## Materials and methods

**Fig. 1:**
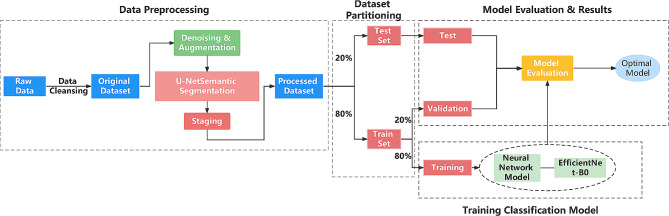
Research Methodology Flowchart

After image augmentation of the datasets, we used the U-NET network for image segmentation. After dividing the data set into the training set, test set, and validation set, we trained the model using the neural network and optimized the model evaluation results to obtain the final model. The implementation details were described in the following sections. Figure 1 was the flowchart of the method in this study.

### Ethics approval

The study was approved by the Medical Ethics Committee of West China Fourth Hospital, Sichuan University (Ethical approval number: HXSY-EC-2,023,042). The study was retrospective. The use of patient information will not adversely affect them, so we waived informed consent, but data confidentiality was ensured. All methods strictly adhered to relevant guidelines and regulations.

### Dataset

A dataset consisting of 498 medical chest radiographs and corresponding clinical cases was obtained from the Department of Radiology of West China Fourth Hospital. The dataset was randomly divided into training and test sets in a ratio of 4:1, as shown in Table 1. And according to the five-fold cross-validation method, each time 20% (i.e., 80 samples) of 398 training sets were randomly selected for validation, and the remaining 80% was used for training. Detailed data regarding specific stages of pneumoconiosis have been prepared and presented in the attached table.

**Table 1:** Summary of data

Data Set	Normal	Stage I	Stage II	Stage III	Total
Training Set	146	117	93	42	398
Test Set	47	30	15	8	100
Total	193	147	108	50	498

The study’s inclusion criteria outlined three main requirements: (1) individuals with a history of dust exposure; (2) patients whose chest radiographs met or exceeded the acceptable quality criteria set out in the GBZ70-2015 guidelines for the diagnosis of occupational pneumoconiosis; and (3) positive cases who had been formally diagnosed with pneumoconiosis and who had obtained diagnostic certificates from qualified units. On the other hand, the exclusion criteria included subjects with pre-existing pulmonary or pleural diseases that would interfere with the diagnosis or grading of pneumoconiosis. These may include but are not limited to, pneumothorax, pleural effusion, or incomplete resection of lung tissue on one side.

### Evaluation indicators

Based on the confusion matrix, the following indicators are commonly used in evaluation models.

**Accuracy** represents the ratio of the number of classifications tested correctly to the total number of tests, calculated as follows.

$$Acc=\frac{TP+TN}{TP+FP+FN+TN}$$

**Recall** indicates the ratio of the number of true positive samples to the actual number of positive samples, calculated as follows.

$$\text{R}\text{e}\text{c}\text{a}\text{l}\text{l}=\frac{TP}{TP+FN}$$

**Precision** indicates the ratio of the number of true positive samples to the number of predicted positive samples, calculated as follows.

$$\text{P}\text{r}\text{e}\text{c}\text{i}\text{s}\text{i}\text{o}\text{n}=\frac{TP}{TP+FP}$$

**F1-score**: In general, we cannot evaluate the classification ability of the model by simply using recall and precision, and we need to combine recall and precision to consider the F1 value, which is the harmonic average of recall and precision. The larger the F1 value, the better the classification ability of the model. The formula is as follows.

$$F1=\frac{2\times \text{R}\text{e}\text{c}\text{a}\text{l}\text{l}\times \text{P}\text{r}\text{e}\text{c}\text{i}\text{s}\text{i}\text{o}\text{n}}{\text{R}\text{e}\text{c}\text{a}\text{l}\text{l}+\text{P}\text{r}\text{e}\text{c}\text{i}\text{s}\text{i}\text{o}\text{n}}$$

**Receiver operating characteristic (ROC)**: The ROC curve evaluates classifier performance across various thresholds and serves as a gauge for classification imbalance. It plots the false positive rate (FPR) on the horizontal axis and the true positive rate (TPR) on the vertical axis. TPR indicates the ratio of the number of true positive samples to the number of all positive samples, while FPR indicates the ratio of the number of false positive samples to the number of all negative samples.

**Area Under Curve (AUC) value**: The AUC curve serves as an indicator to assess classifier performance by illustrating its capability to accurately classify positive and negative samples across various thresholds. A higher AUC value, approaching 1, indicates superior classifier performance, while a value closer to 0.5 suggests the classifier’s performance is akin to random classification.

**Quadratic weighted kappa (QWK)**: QWK is an indicator used to measure the consistency of classifiers. It considers the consistency between the predicted results and the actual results, and weights the degree of error. The value range of QWK is usually from − 1 to 1, where 1 represents complete consistency, 0 represents consistency with random selection, and negative numbers indicate lower consistency between predicted and actual results than random selection. The formula is as follows.

$$\kappa =1-\frac{\sum _{i,j} {w}_{i,j}{O}_{i,j}}{\sum _{i,j} {w}_{i,j}{E}_{i,j}}$$

QWK provides a more comprehensive measure of model accuracy relative to accuracy (Acc). For example, misclassifying normal as pneumoconiosis I has the same effect on accuracy as misclassifying normal as pneumoconiosis III, but the latter is clearly the more serious error. QWK will produce a greater decrease in the latter, which makes evaluating the model more comprehensive.

### Image preprocessing

**Fig. 2:**
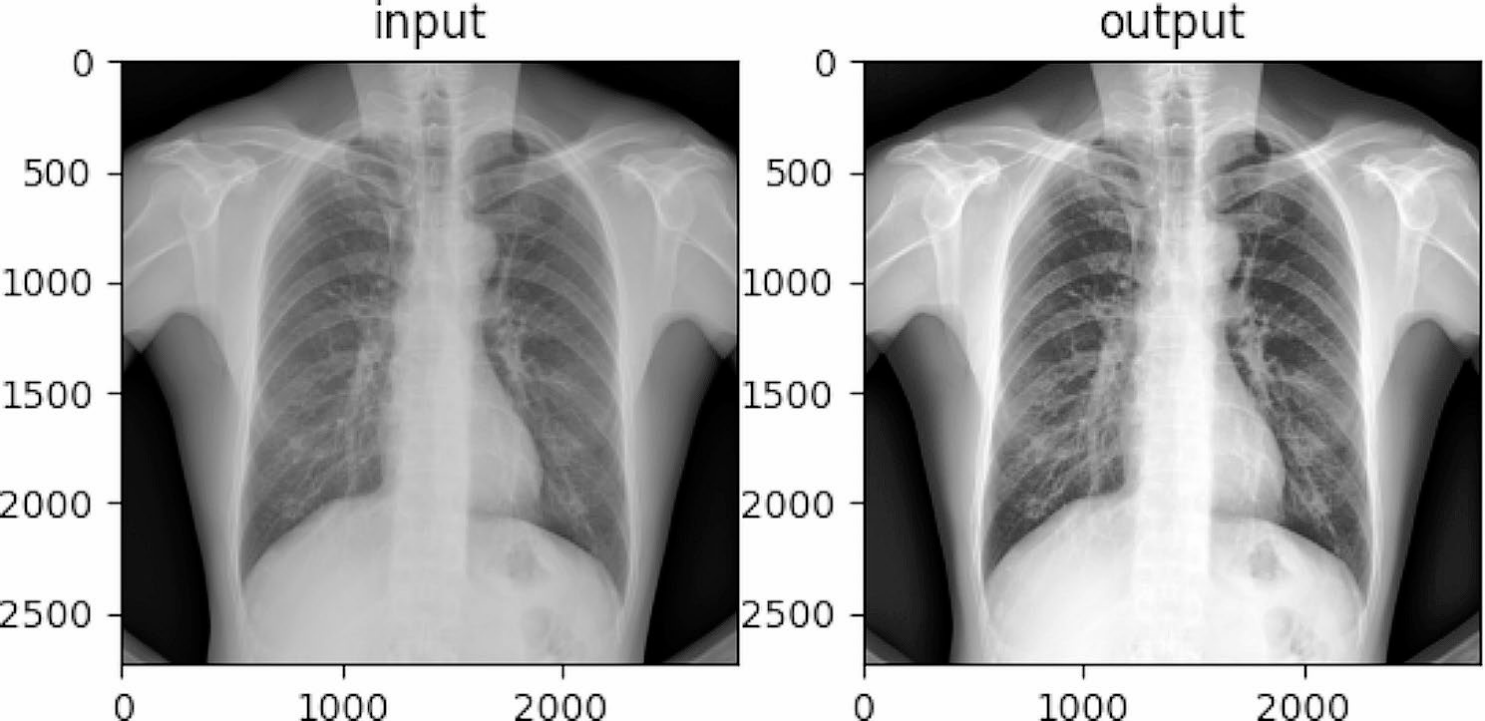
Histogram equalization before and after

Use histogram equalization to enhance the image. Since some of the tissue structure information in an X-ray chest film may not have significant contrast or have subtle gray level differences from the surrounding area, to highlight the target area or object, we need to adjust the gray level of the image to emphasize the contrast difference between the target area or object and its surroundings. The histogram equalization algorithm [19] enhances the image contrast by redistributing the number of different pixel gray levels in the image so that the number of pixels in each gray level is equal. For medical images such as chest radiographs of pneumoconiosis, histogram equalization can enhance the clarity and differentiation of lesions and help doctors accurately diagnose and treat patients. The effect was shown in Fig. 2.

### Image segmentation

Since the resolution of the chest X-ray of pneumoconiosis after image preprocessing is larger (2980 × 2980) and there are multiple types of targets in the X-ray, which will affect the training of the classification network, it is still not suitable for the direct use of the neural network that can accomplish the classification task. Therefore, it is necessary to use image segmentation to segment the chest X-ray image of a pneumoconiosis patient into multiple regions and train classification on each region. This can simultaneously increase the number of samples and reduce the computational complexity. According to the selected image segmentation method, we used the U-Net semantic segmentation method with better results. This model is based on convolutional neural networks and is widely used in the field of image segmentation.

#### U-net image segmentation

U-Net has an efficient architecture designed for biomedical image segmentation tasks, featuring a contracting path for capturing context and a symmetric expanding path for precise localization. This design enables efficient use of limited training data and facilitates accurate segmentation even with small datasets [20]. Additionally, skip connections between contracting and expanding paths aid in preserving spatial information [21].

We used image segmentation to exclude irrelevant parts of the chest radiographs and reduce the image resolution, with a total of 1734 pairs of images and masking layers to train the U-Net model.

**Fig. 3:**
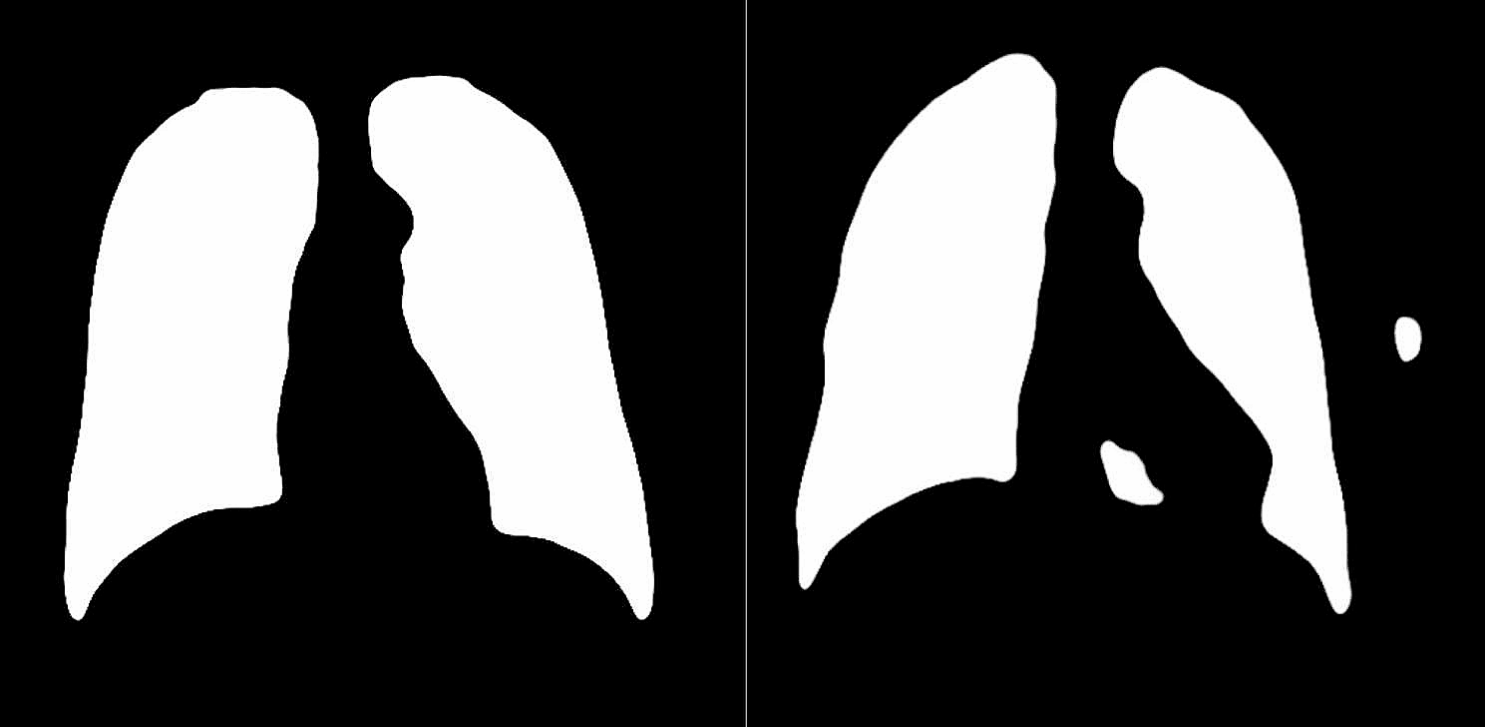
A complete mask layer (left) and a defective mask layer (right)

The mask layer obtained by U-Net was shown in Fig. 3, and most of them were complete, as shown in the left figure. But a few had small defects as shown in the right figure. So further image processing is required to remove the defects and get a complete mask layer [22].

#### Morphological manipulation of the mask layer

These small defects can be removed by performing “open-close” morphology operations on the above defective mask layers with appropriately sized convolutional kernels. The meanings of open, close, erode and dilate in OpenCV-Python are respectively:

- Erode: convolution of the image by a certain size of convolution kernel, causing the white (transparent) parts to shrink.
- Dilate: the image is convolved with a convolution kernel of a certain size and the white (transparent) part is dilated.
- Open: erode before dilating.
- Close: dilate before eroding.

**Fig. 4:**
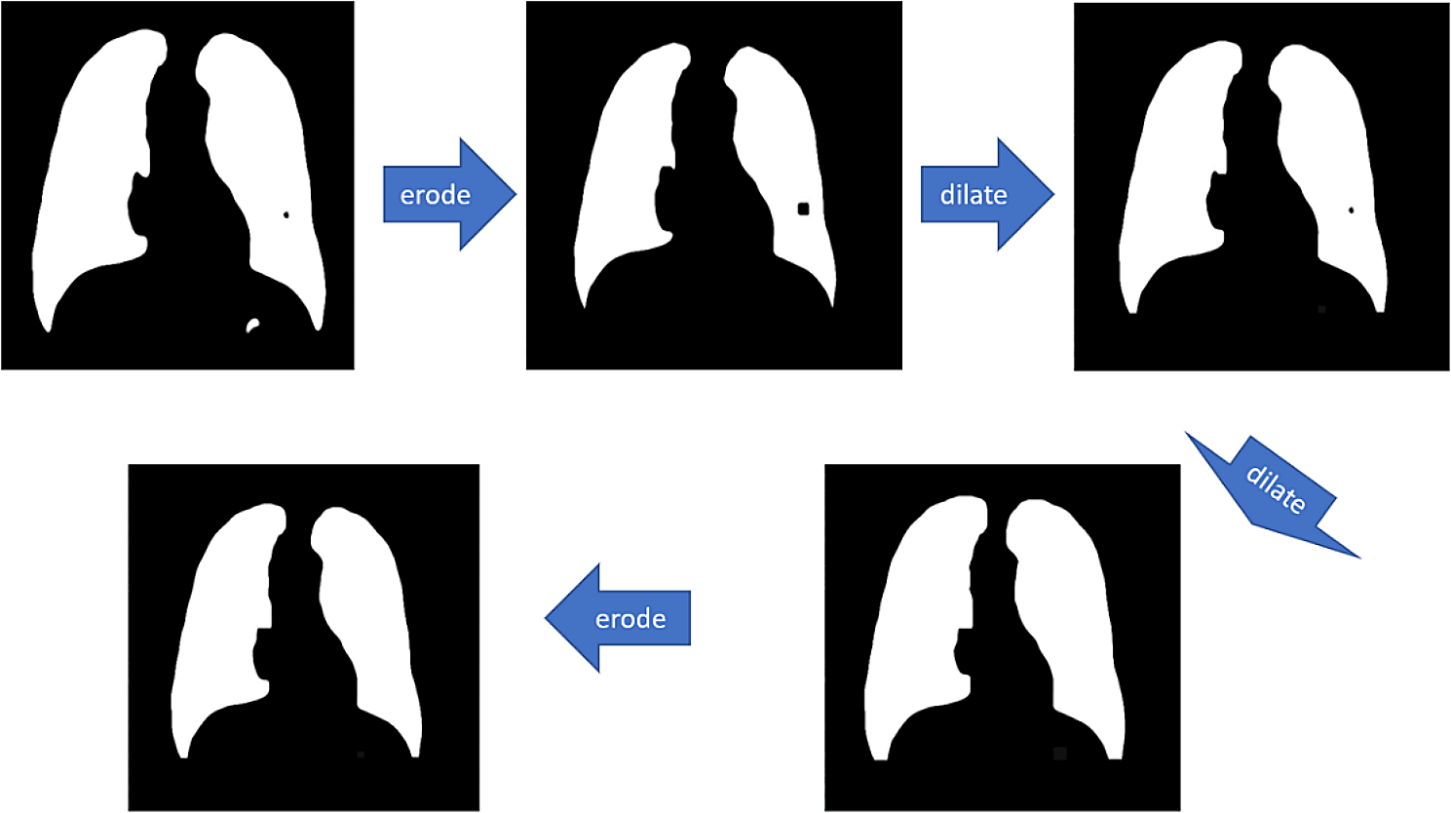
Schematic diagram of mask layer processing

The specific process is shown in Fig. 4:

Morphological operator size is determined by two parameters:

- number of contours: normal pneumoconiosis pictures have only two contours, but for defective pneumoconiosis pictures there are usually more than two contours.
- fragmentation factor: the ratio of the total perimeter of the contour L to the total area of the contour S.

$$\lambda =\frac{{\sum }_{i=1}^{N}{L}_{i}}{{\sum }_{i=1}^{N}{S}_{i}}$$

where ${L}_{i}$ and ${S}_{i}$ denote the perimeter and area of the i.th segment profile, respectively. The larger the fragmentation coefficient, the more fragmented the dusty lung picture is. And the details were shown in Table 2. Additionally, the morphological operators smooth the segmentation mask. To mitigate potential adverse impacts, we employed the smallest possible morphological operator that still effectively eliminated imperfections. This approach ensured minimal alteration to the mask while maintaining its integrity. Larger morphological operator like nine pixels, while effective in eliminating imperfections, resulted in an excessively smooth mask, potentially compromising the original information.

**Table 2:** Guidelines for selection of morphological size (in pixels)

Number of contours	Fragmentation factor (/$1{0}^{-2}$)	Morphological operator size (px)
2	Arbitrarily	None
2–5	< 3.2	3
2–5	3.2-4	5
2–5	> 4	7
> 5	Arbitrarily	9

**Fig. 5:**
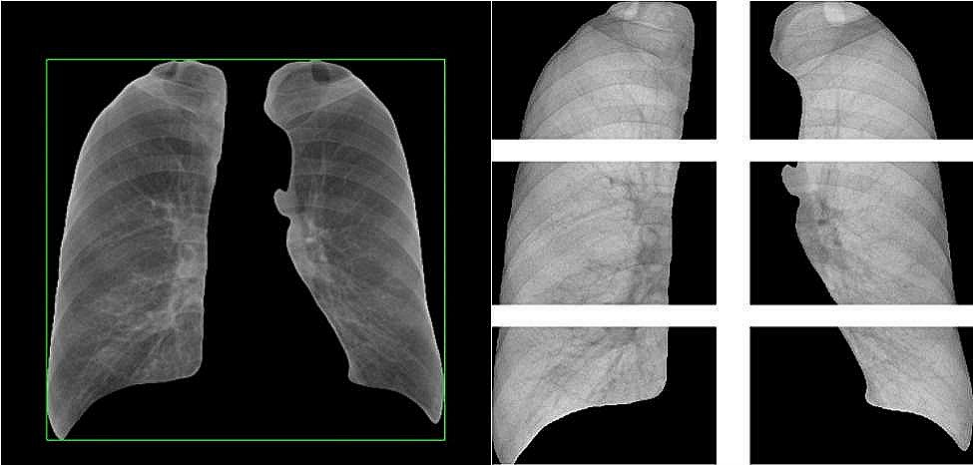
Schematic diagram of image partitioning

After obtaining the complete mask layer, the mask layer was acted on the original chest film to separate the lungs from other parts and find out the outer rectangle of the mask layer; then according to the national standard of chest film classification, the chest film can be divided into six parts: upper-left, upper-right, middle-left, middle-right, lower-left and lower-right. Divide the above outer rectangle of the chest film into six small rectangles to get six partitions, as Fig. 5 shown.

The labeled dataset obtained in this study was independently subjected to the first round of reading by radiologists of post-partitioned chest radiographs by the national standard GBZ70-2015, and the staging stage of pneumoconiosis was determined by independently annotating the abundance of small turbidities (grades 0, 1, 2, or 3) and the presence of large turbidities for each subregion, after which we trained the dataset.

### Data enhancement

After the above preprocessing, the resolution of the chest X-ray film was still too large, and it would take a long time to load and process if it was directly inputted into the Convolutional Neural Networks (CNN), considering the memory limitations of the device and the convenience of the CNN inputs, the above-processed image was down-sampled to 1000 × 1000.

Data enhancement such as rotation and cropping are a desirable approach to effectively expand the training samples and enhance the model generalization and robustness performance while suppressing overfitting. After testing, it was found that mild data enhancement by small angle (5^°^~10^°^) rotation, small range (110%~120%) zoom, and horizontal flipping could improve the accuracy, but after too extensive data enhancement (including rotation, horizontal flipping, vertical flipping, Gaussian noise, etc.), the accuracy of the model decreased, and the results were shown in Table 3.

**Table 3:** Accuracy of efficient-net after using different image enhancements

Data Enhancement	Staging Accuracy
No data enhancement is used	80%
Use light data enhancement	83%
Overused data enhancements	77%

We used mild data augmentation on the data after partitioning with U-Net before training the staging model.

### Efficient-net-based staging of pneumoconiosis

**Fig. 6:**
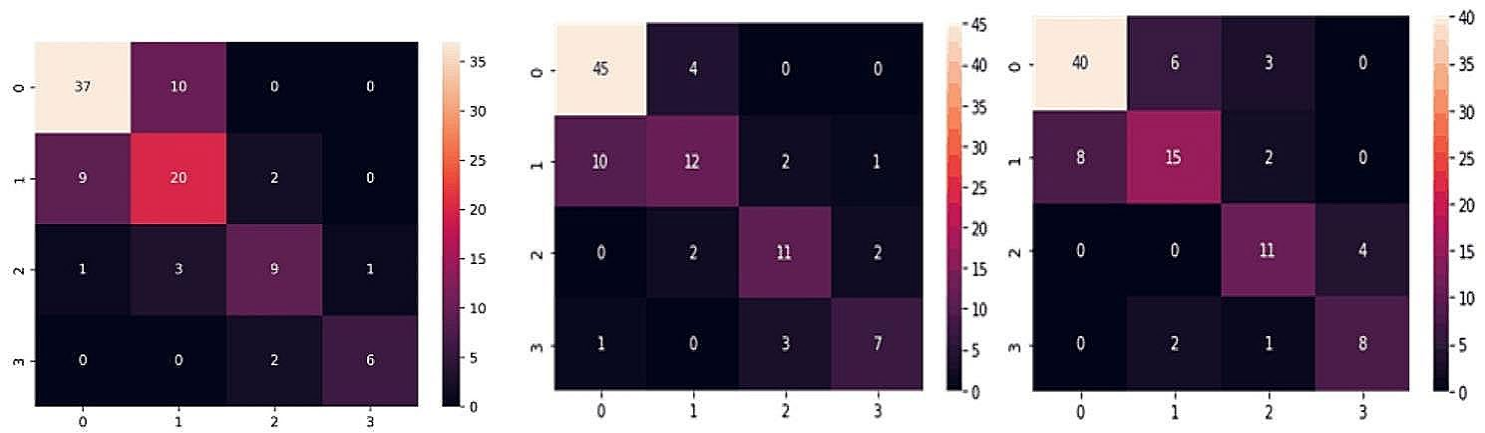
Confusion matrix of ResNet18, MobileNet-V2, and EfficientNet-B0-V2 models (from left to right) on the validation set

We have trained pneumoconiosis staging using neural networks with different structures such as VGG16, ResNet18, Mobile-Net, Efficient-Net, etc. The accuracy and Quadratic Weighted Kappa (QWK) obtained based on the corresponding confusion matrices (Fig. 6) were compared with the EfficientNet-B0-V1 model as shown in Table 4. The EfficientNet-B0-V1 works better because Efficient-Net has elastic structures such as scalable convolutional kernel, so it has good performance for large images, such as X-ray film.

**Table 4:** Comparison of accuracy and QWK of efficient-net with other models

Neural Network	ACC	QWK
ResNet18	72%	0.822
MobileNet-V2	75%	0.833
EfficientNet-B0-V2	74%	0.813
EfficientNet-B0-V1	83%	0.889

The overall accuracy of a whole image can be obtained by combining the diagnostic results of each partitioning by Table 5.

**Table 5:** Pneumoconiosis classification scale

	Description
Normal	No opacities discover, or Level 1 profusion of opacity presented in one subregion
Stage I	Level 1 profusion of opacities presented in more than two subregions, or Level 2 profusion of opacities presented in four subregions or less
Stage II	Level 2 profusion of opacities presented in four subregions or more, or Level 3 profusion of opacities presented
Stage III	Large opacities presented

Efficient-Net has three main parameters: width ($\omega$), depth (d) and resolution I. The width represents the number of convolutional layers or channels; the depth represents the number of layers of the network after each convolution; and the resolution represents the size of the input image.

For how to balance the three dimensions of resolution, depth and width to achieve the optimization of convolutional networks in terms of accuracy and efficiency, Efficient-Net proposes a model composite scaling method (composite scaling method), which envisions that in a basic network, the network can be scaled up in the dimensions of width, depth and resolution, and the main idea of Efficient-Net is to synthesize these three dimensions for composite scaling of the network.

**Fig. 7:**
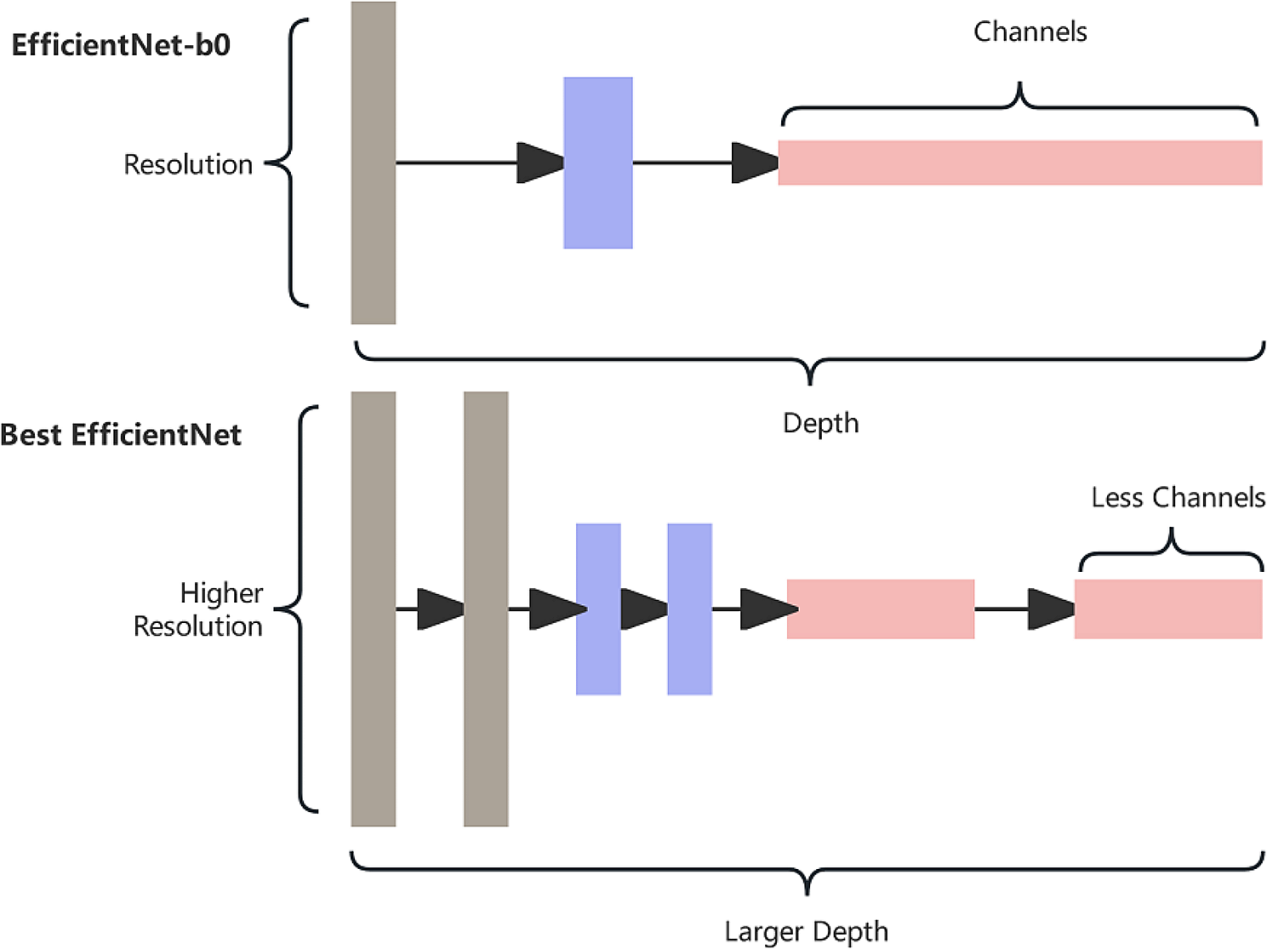
Comparison of EfficientNet-B0 and Efficient-Net used in this paper

The unsegmented pneumoconiosis dataset had only one class of objects: dust lungs. As a result, the images had few features, so our model didn’t need many convolutional kernels to generate many channels for feature extraction. Therefore, we chose Efficient-Net with a small width; moreover, the segmented pneumoconiosis dataset still had a large resolution (1000 × 1000). Therefore, we needed a CNN with larger depth to process larger images. The basic structure of the model was shown in Fig. 7.

After repeated experiments, three hyperparameters: depth factor, width factor, and resolution are selected by grid search method. We finally found that depth factor = 2, width factor = 0.5, and Resolution = 1000 was the optimal choice (relative to the factors in EfficientNet-B0).

### Multi-stage combined staging diagnosis

Clinical practice shows that the diagnosis of pneumoconiosis stage III is the easiest, the distinction between stage I and stage II is the second, and the distinction between stage 0 and stage I is the most difficult.

Following the assessment and analysis, the Efficient-Net model has been identified as having a challenge in differentiating between stages I and II in multi-classification. To enhance the accuracy of the model, a multi-stage joint method has been employed.

#### Stage 1

Distinguish between stage 0 patients and stage I/II/III patients;

#### Stage 2

Distinguish between stage I/II and stage III patients;

#### Stage 3

Distinguish between stage I/II patients.

**Fig. 8:**
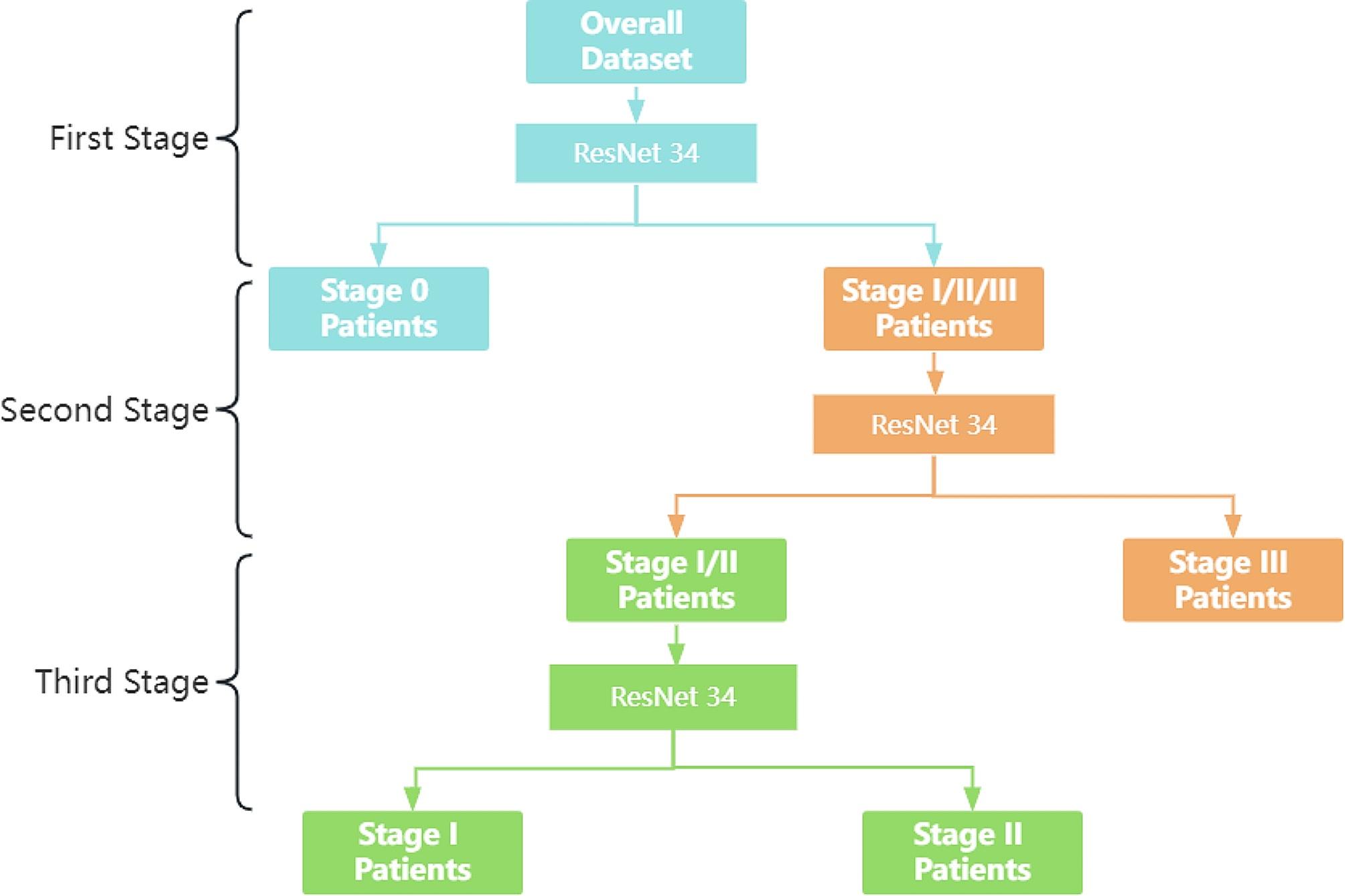
Multi-stage joint flow chart

The flowchart was shown in Fig. 8:

Through the joint multi-phase approach, a more specialized model can be trained, and the generalization ability of the model can also be increased, increasing its robustness. When higher sensitivity of the model is needed to distinguish between stage 0 and 1/2/3, and higher specificity of the model is needed to distinguish between stage 1/2, the characteristic improvement of Res-Net34 for each stage according to the distinguishing characteristics of the stages can bring out the advantages of the model and improve the accuracy.

### Experimental setup and model training

The neural network models for the multi-stage joint staging model were trained separately. A five-fold cross-validation method was used to optimize the network parameters in the training set, and then the model with the highest accuracy was selected for testing in an external test set. Its training environment was NVIDIA RTX 3060 GPU (8GB), and all code was implemented by Python 3.9.8. The batch size was initially set to 32, the optimizer was Adam, the weights were initialized using default initializer (standard normal distribution), and the initial learning rate was set to 0.0001. We used a stepped learning rate tuning strategy, where the learning rate was tuned to 1/10th of the original rate every 15 calendar hours, and training was stopped after 1000 iterations.

Convolutional layers use an $ReLU$ activation function, which is a function that is semi-corrected from the bottom, and the mathematical formula is specified as follows:

$$f\left(x\right)=\text{m}\text{a}\text{x}(0,x)$$

Where, the$x$ denotes the input. Staging pneumoconiosis is a multiclassification problem. For the classification problem, the most used loss function is the Cross Entropy Loss, which can be expressed as.

$${L}_{CE}=-\sum _{i=1}^{4} {y}_{i}\text{l}\text{o}\text{g}\left({p}_{i}\right)$$

where.$4$ is the number of pneumoconiosis staging;${y}_{i}$ is a One-hot vector, the outputs on the staging are 0 except for the target staging which is 1;${p}_{i}$ is the prediction of the neural network, that is, the staging $i$ probability of the staging.

Sometimes we may only want to distinguish whether we are sick or not, which is a binary classification problem. In this case, the network predictions end up with only 2 categories, assuming that for each category the probability of prediction is respectively $p$ and$1-p$, then at this point the cross-entropy loss function is formulated as:

$${\text{L}}_{CE}=-[y\cdot \text{l}\text{o}\text{g}(p)+(1-y)\cdot \text{l}\text{o}\text{g}(1-p\left)\right]$$

Where $y$ is the sample label, positive sample label is 1, and negative sample label is $0. p$ denotes the probability of predicting a positive sample.

## Results

### U-net semantic segmentation

**Fig. 9:**
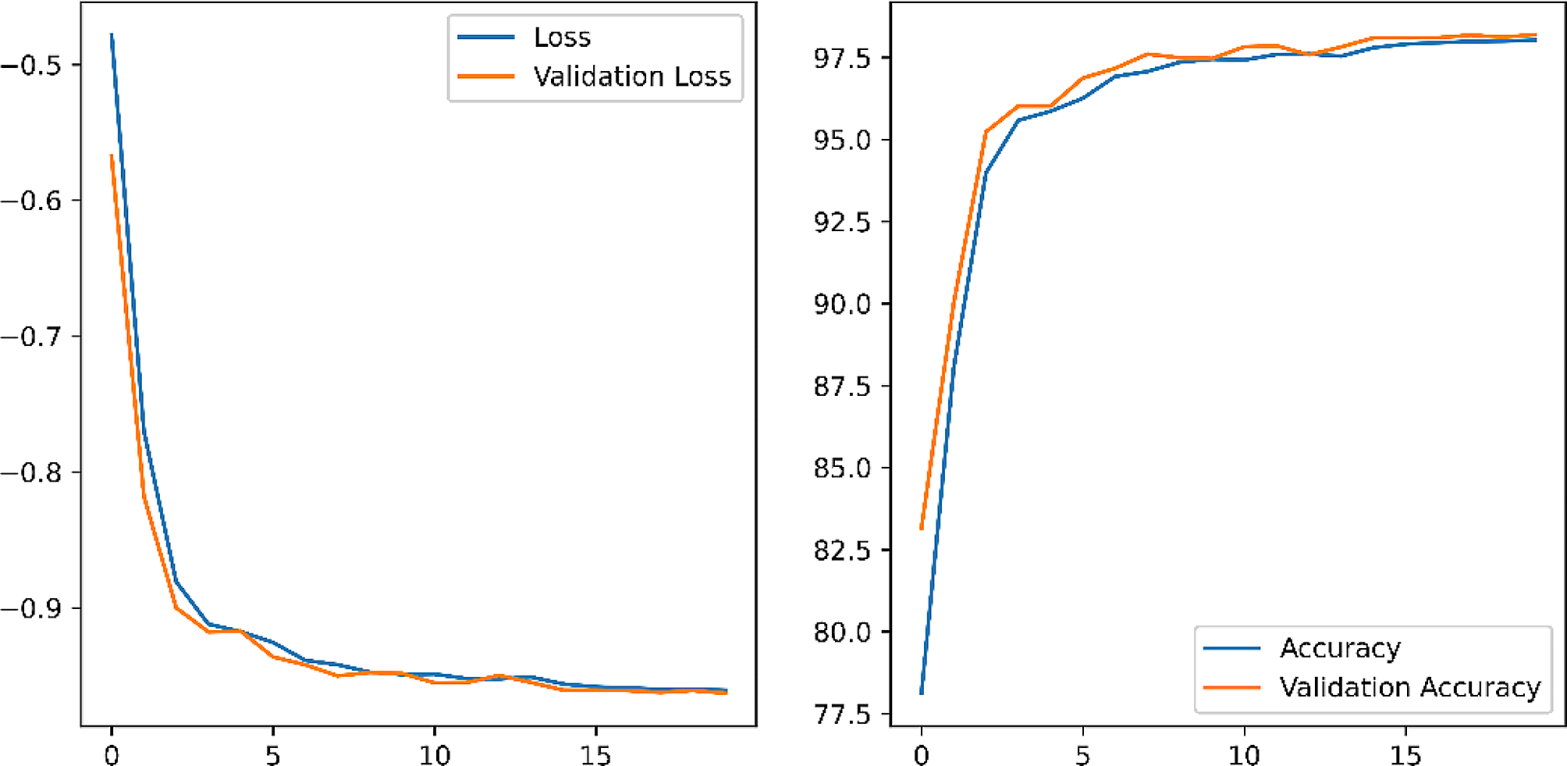
Variation of loss and accuracy with the number of training sessions

The U-Net semantic segmentation model evaluation utilized an open-source dataset derived from Kaggle containing training and test chest slices and already labeled mask layers. After 20 cycles of training, the localization accuracy rate IoU (Intersection over Union) reached 97.8%. Figure 9 showed the trend of loss function and accuracy with the number of training cycles, respectively.

**Fig. 10:**
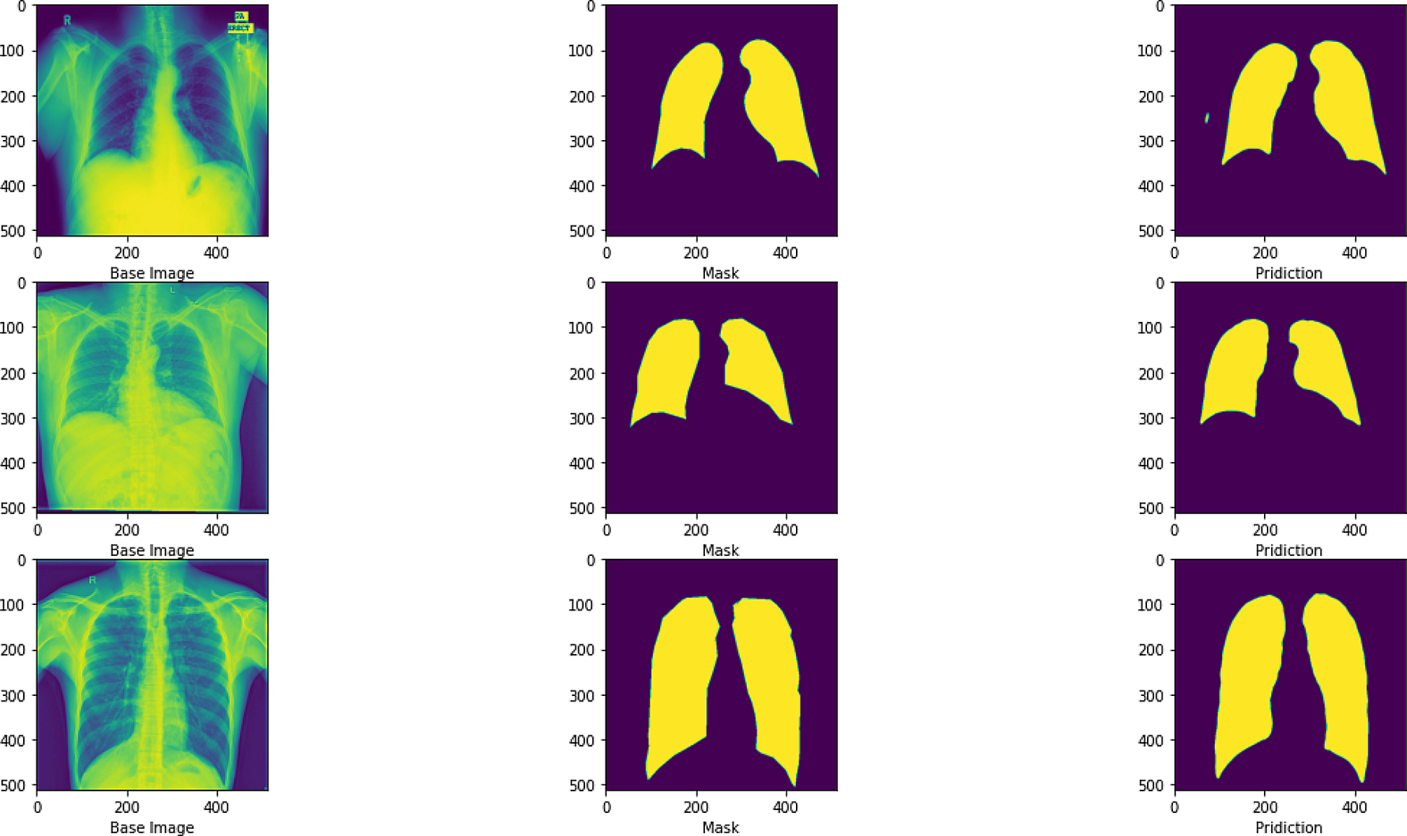
U-net image segmentation effect diagram

The loss degree and validation set loss degree fit was high, indicating that there was no overfitting, as shown in the left figure. The accuracy of both the training and validation sets increased with the increase of cycles, as shown in the right figure. This indicated that the model was well trained, and the final accuracy gradually converged to about 97.8%. Figure 10 showed the effect of U-Net image segmentation, and it can be seen from the figure that the accuracy of the U-Net image model was high.

### Efficient-net multi-classification model evaluation

By referring to other neural network models in the field of medical images, this study used the accuracy, precision, recall, F1-score, quadratic weighted kappa score (QWK) and ROC curve and AUC to evaluate the performance of the model. All the above metrics except ROC, can be calculated by the confusion matrix. A confusion matrix is used as a table for evaluating the performance of a classification model, where each row represents the actual category, and each column represents the predicted category. Table 6 showed the visualization of the classification results on the validation set for the multi-classification model applying the neural network Efficient-Net.

**Table 6:** Confusion matrix of efficient-net multi-classification model on validation set

Predict Reality	Normal	Stage I	Stage II	Stage III	Total
Normal	44	3	0	0	47
Stage I	7	20	3	0	30
Stage II	0	3	12	0	15
Stage III	0	1	0	7	8
Total	51	27	15	7	100

The above confusion matrix showed that the model accuracy was 83% and most of the data was distributed on the main diagonal, which indicated that the model was accurate.

The precision, recall, and F1-score of each stage were shown in Table 7.

**Table 7:** Accuracy rates by stage

	Normal	Stage I	Stage II	Stage III
Precision	0.86	0.74	0.80	1.00
Recall	0.94	0.67	0.80	0.88
F1-score	0.89	0.70	0.80	0.93
QWK	0.889			

### Multi-stage combined staged diagnostic assessment

The multi-stage joint staging diagnostic model integrated multiple neural network models to have higher accuracy, and its confusion matrix was shown in Table 8.

**Table 8:** Confusion matrix for the multi-stage combined staging diagnostic model

Predict Reality	Normal	Stage I	Stage II	Stage III	Total
**Normal**	130	1	0	0	131
**Stage I**	9	9	4	1	23
**Stage II**	1	1	17	4	23
**Stage III**	0	0	1	15	16
**Total**	140	11	22	20	193

The accuracy of the multi-stage joint staging diagnostic model for each partition was shown in Table 9:

**Table 9:** Partition accuracy

Subregion	Dichotomous (distinguishing only between the presence or absence of disease)			Staging Accuracy
	Accuracy	Sensitivity	Specificity	
Top-right	0.929	0.882	0.942	0.866
Top- left	0.912	0.829	0.930	0.864
Middle-right	0.934	0.908	0.941	0.895
Middle-left	0.881	0.918	0.883	0.932
Bottom-right	0.856	0.604	0.907	0.849
Bottom-left	0.818	0.500	0.876	0.895
Subject	0.973	0.981	0.970	0.886

The accuracy of the whole chest radiograph was obtained after aggregating the partitioned accuracies and calculating them according to the criteria above. The model of the precision, recall, and F1-scores for each staging were shown in Table 10:

**Table 10:** Accuracy rates by staging

	Normal	Stage I	Stage II	Stage III
Precision	0.93	0.82	0.77	0.79
Recall	0.99	0.41	0.74	0.94
F1-score	0.96	0.55	0.76	0.86
QWK	0.94			

**Fig. 11:**
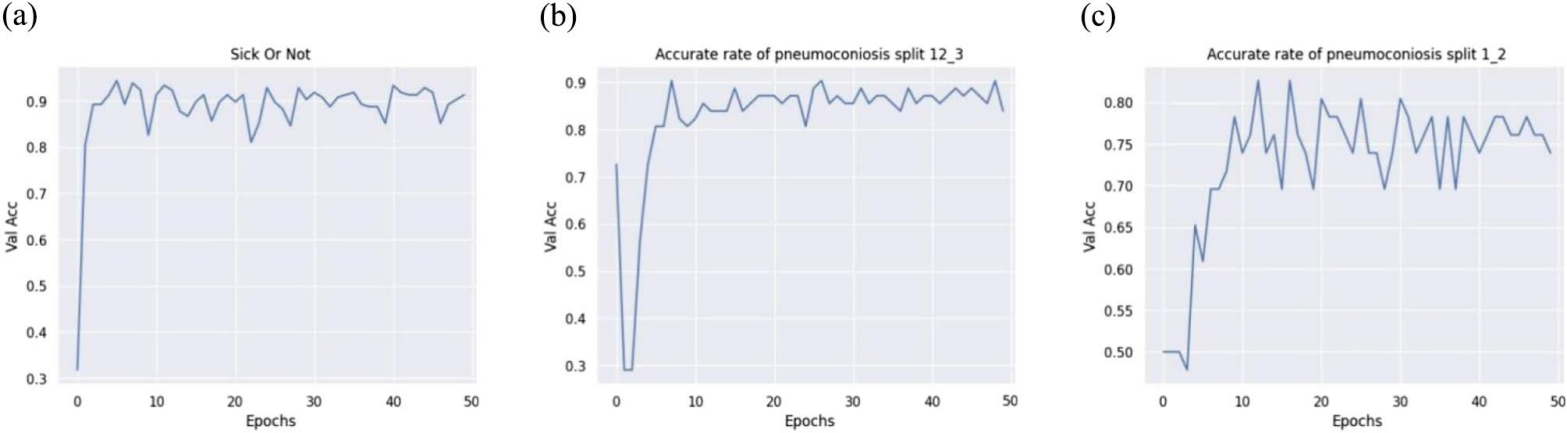
Figure 11 Variation of accuracy with epoch. (**a**) Distinguish between Stage 0 and Stage I/II/III. (**b**) Distinguish between Stage I/II and Stage III. (**c**) Distinguish between Stage I and II

The accuracy of the model on the validation set varied during the model training process as shown in Fig. 11:

Comparing Efficient-Net single-model multi-classification and multi-stage joint staging diagnostic models, the comparison of their indicators were as Table 11 showed that the multi-stage joint staging diagnostic model had made great progress compared with Efficient-Net multi-classification.

**Table 11:** Comparison of efficient-net multi-classification model and multi-stage joint staging

Efficient-Net Single-model Multi-Classification				Multi-stage joint Staging Diagnostic Model			
Dichotomous			Staging Accuracy	Dichotomous			Staging Accuracy
Accuracy	Sensitivity	Specificity		Accuracy	Sensitivity	Specificity	
90.0%	86.8%	93.6%	83.0%	94.3%	84.1%	99.2%	89.0%

**Fig. 12:**
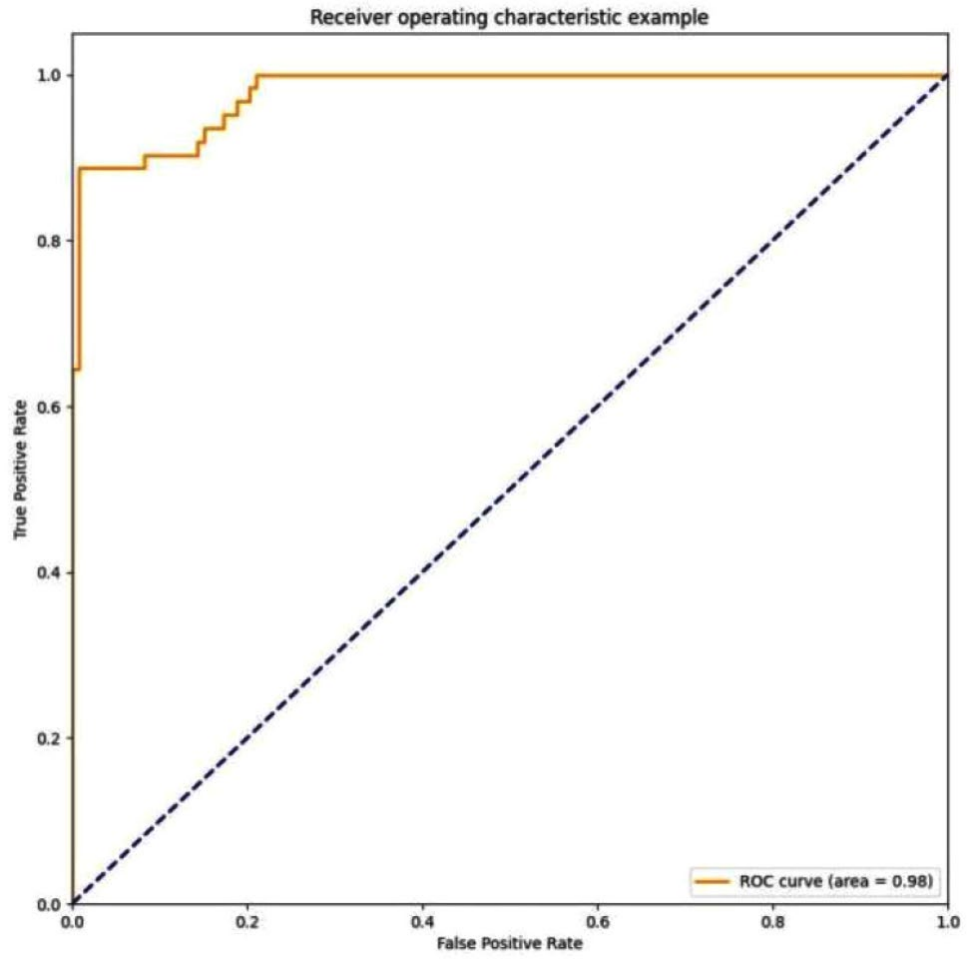
ROC curves for the multi-stage combined staging diagnostic model

The dichotomous ROC curves for the multi-stage combined staging diagnostic model were shown in Fig. 12. The differentiation effect of the model was excellent.

### Stability analysis

**Fig. 13:**
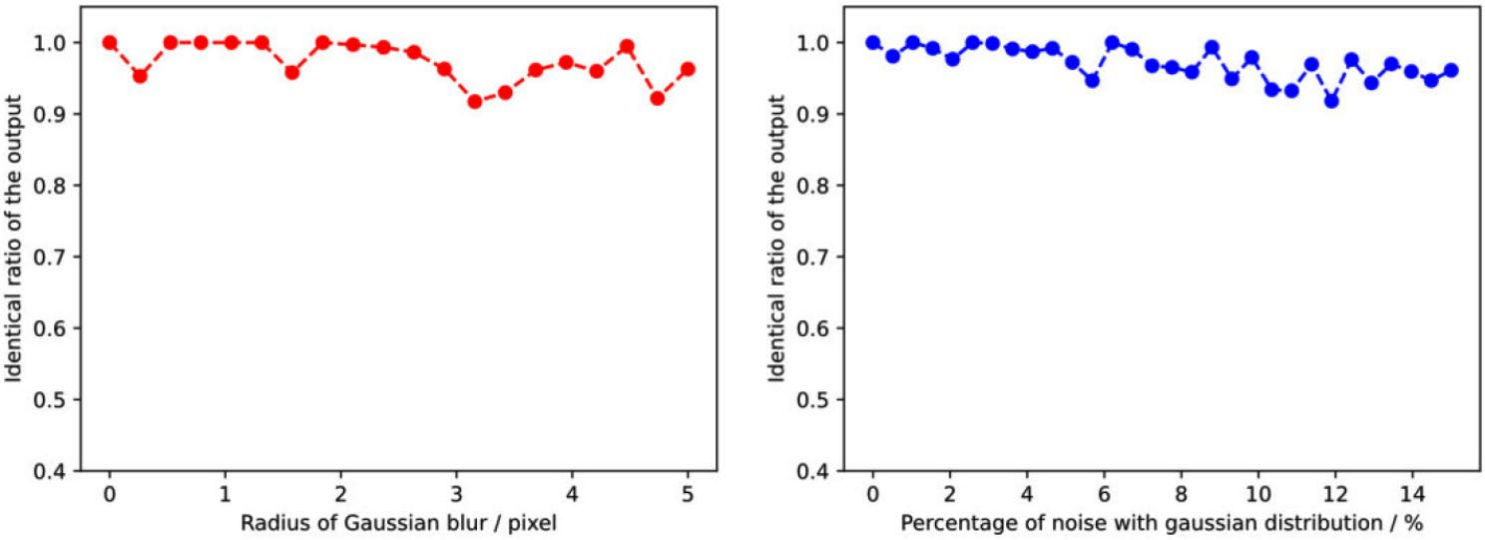
The consistency of the model output on the test set

In pneumoconiosis clinical diagnosis, we expect the output of neural networks to be reliable and consistent. If the network performs unstable when the input data changes slightly or with slight perturbations, its reliability will be threatened. Stability analysis can help us to understand the sensitivity of the network to the input data and take corresponding measures to improve the reliability of the network. To test the stability of the model, we added Gaussian blur with different radii (units: pixels) and different proportions of Gaussian noise to the model. The larger the blur radius, the stronger the blur. The higher the noise percentage, the more noise. Figure 13 showed that the result of testing the consistency of the model output on the test set. It can be observed that the consistency of the model decreased slightly with increasing blur and noise, but still remained at a higher level. Therefore, the model can be considered to have high stability.

## Discussion

In the diagnosis of pneumoconiosis, the relevant occupational history, the condition of the patient and the X-ray chest film of the patient are the main evidence for the doctor to determine whether the patient is suffering from pneumoconiosis and to diagnose the specific pneumoconiosis stage. Due to the complexity of the human body structure and organization, X-ray chest radiographs are characterized by poor contrast, wide dynamic range and rich details, etc. Primary healthcare institutions are severely limited by the bottleneck of capacity and talent, especially the diagnostic decision-making ability of healthcare institutions in rural areas, which makes it easy for inconsistent and unreliable diagnostic results to occur. Therefore, for the application of pneumoconiosis diagnosis, doctors need an automated auxiliary diagnostic system to help them complete the diagnosis of pneumoconiosis, to reduce the difficulty of reading radiographs and improve diagnostic efficiency. In this study, we utilized powerful neural network learning and well-labeled chest radiographs to develop a deep learning-based pneumoconiosis staging diagnostic model.

The main difference between the joint deep learning-based multi-stage model proposed in this study and other models for pneumoconiosis screening is:

1. Compared with other machine learning methods, our binary classification result achieved 94.3% accuracy, which is significantly better than other results [23], where the best result among general neural network classifiers is only 83%.
2. The model we developed had higher screening accuracy in the paper reports on the staged diagnosis of pneumoconiosis. In the study of Yang F [17] et al., the overall accuracy of the four classifications was only 70.1%, and the model screening accuracy in the study of Cai [6] et al. was 79%, even though the AUC for experienced physicians was only 0.668 and 0.772 [24]. Our model not only diagnoses the disease, but also focuses on staging the diagnosis, with an accuracy of 83%.
3. After we use the improved U-net neural network for image segmentation, we use Efficient-Net to train the model with fewer parameters and higher accuracy compared to other models. Overfitting is avoided by adding Dropout layer and L2 regularization, which improves the model generalization ability. Efficient-Net optimizes the use of computational resources by systematically balancing the depth, width, and resolution of the network, which enables more efficient use of hardware resources such as memory and processing power.
4. Compared with our previous research [15], the image segmentation method used in this study is U-Net, with an IOU of 97.8%, which is better than the previous 94.3%. Although the accuracy (89%) is slightly lower than the previous 90.4%, considering that the sample size of the previous study was more than twice that of this study, increasing the sample size may increase the accuracy. The previous study’s AUC was 0.96, but in this study, the AUC increased to 0.98. In addition, this study added new evaluation indicators, QWK which equals 0.94, indicating that the classification model has excellent performance.
5. In terms of methods, Liton [25] et al. employs an integrated model for classification using weighted average and majority voting, but only for binary classification. Yang F [17] et al. first segments the lungs from the background using U-net and then classifies them using Res-Net, also only for binary classification. Liuzhuo Zhang [18] divides the lung into six regions using U-net, classifies each partition using Res-Net with four classifications, and finally obtains the radiograph’s overall result. Zhang [26] initially divided the lung into six regions using U-net and then staged it using traditional algorithms. In contrast, we utilized a segmentation method prior to classification. During the segmentation stage, we first separated the lungs from the background using U-net. We then repaired any defects in the mask through morphological manipulation before dividing the lungs into six regions. This approach achieved more accurate partitioning and avoided any defects in the mask that could affect the classification. In the classification stage, we utilized two models. The first is Efficient-Net, which is simpler, faster, and more accurate for direct classification. The second is Res-Net, which is used for multi-stage classification and further improves accuracy.

Our research results showed that for single-stage and multi-stage models, the QWK of the single-stage model was 0.89, and the QWK of the multi-stage model was 0.94. The accuracy of the single-stage model was 83%. The multi-stage four classification was 89%, and the accuracy of the multi-stage two classification was 97.3%, which proved that Multistage Joint Staging Diagnostic Model outperformed the Efficient-Net Single model Multi Classification.

The advantage of QWK lies in its sensitivity to the severity of misclassifications: Accuracy treats all misclassifications equally, regardless of their severity. But QWK will consider the degree of consistency between the predicted results and the actual labels, and conduct a more detailed evaluation of the model performance by imposing stricter penalties on larger differences. For example, when the model misclassifies the 0 stage of a chest X-ray as stage 1 and stage 3, its accuracy is the same, but for QWK, the latter is more severe than the former.

The high resolution of digital chest radiographs and the often-limited computational resources available in practice make it unfeasible to develop and deploy neural networks for direct use on full chest radiographs. This necessitates either downsampling the images or reducing the model capacity to a manageable size. However, since the diagnosis of pneumoconiosis heavily relies on recognizing subtle image features, it is imperative to maintain image resolution and feature richness without compromise. Consequently, in this study, we optimized the model while reducing the demand for computational resources by partitioning the chest radio graph and learning the sub-partitions for classification and then overall classification and diagnosis. This reduced the computational volume and increased the actual training sample size. Given the limited amount of pneumoconiosis data, data augmentation is employed to enhance model generalization. Augmenting the dataset with variations of the original images, such as rotations, flips, shifts, and zooms, exposes the model to a broader range of possible input variations, thereby reducing overfitting and improving its ability to handle unseen data. However, excessive data augmentation can reduce accuracy, as inappropriate image transformations may distort the fundamental pathological features of pneumoconiosis. Furthermore, in the image segmentation stage, morphological operations were employed to rectify deficiencies in the mask. This was undertaken to preclude any adverse impact on the classification process. Additionally, two methodologies, Efficient-Net and Multi-stage Classification Model, were subjected to testing and comparison. The classification was conducted on each segment, and the results were subsequently aggregated to yield the outcome for the entire image.

There were some limitations in this study. Firstly, the training set data was relatively small, and the samples were unevenly distributed due to practical constraints, especially the small number of patients with stage II and III pneumoconiosis. The sample size needs to be enlarged to increase the data to optimize the model. In the future, we will collect more data on stage III pneumoconiosis patients to enhance the balance of the data. From an epidemiological point of view, all images were collected from a single hospital, which may warn against the generalization ability of the proposed method. Therefore, it is strongly recommended that future studies consider merging data from multiple centers to verify its generalizability to different conditions. Secondly, the current dataset contained only chest radiographs. Although X-ray chest radiographs are the gold standard for pneumoconiosis diagnosis, they lack detailed details of turbid areas in the lungs, which can be improved by computed tomography (CT) images, and therefore the inclusion of CT tests should be considered in future diagnostic studies on pneumoconiosis staging. Finally, due to the special characteristics of radiographs, it is difficult to use data enhancement, and image enhancement can only be performed by using minor adjustments such as horizontal flip, small magnification, and small-angle rotation, which have little effect on alleviating overfitting, so more extensive image enhancement is needed for effective preprocessing to enrich the diversity of data and avoid overfitting.

### Electronic supplementary material

Below is the link to the electronic supplementary material.

Supplementary Material 1

## Data Availability

The data that support the findings of this study are available from West China Fourth Hospital, but restrictions apply to the availability of these data, which were used under license for the current study, and so are not publicly available. Data are however available from the corresponding authors upon reasonable request.
