# Supplementary material for: Deep learning pneumoconiosis staging and diagnosis system based on multi-stage joint approach
Source: BMC Med Imaging. 2024 Jul 2;24:165. doi: 10.1186/s12880-024-01337-x (PMC11221180; doi:10.1186/s12880-024-01337-x)
Supplement: Supplementary file 1 — Supplementary Material 1 [file 12880_2024_1337_MOESM1_ESM.docx]

**Supplemental material**

This is the supplementary material for the revision that we didn’t submit to the system last time. It is a response to the previous revision. In order to ensure the reproducibility of the results of the paper, the hyperparameters of the U-net, EfficientNet model and multi-stage ResNet model during our training process are listed in the following tables.

U-net：

| Learning rate | 2e-4 |
| --- | --- |
| Weight decay | 1e-4 |
| Batch size | 16 |
| Number of epochs | 100 |
| Activation function | ReLU |

EfficientNet

| Learning rate | 5e-5 |
| --- | --- |
| Weight decay | 1e-4 |
| Batch size | 32 |
| Number of epochs | 100 |
| Activation function | ReLU |

ResNet

| Learning rate (stage 1) | 4e-5 |
| --- | --- |
| Learning rate (stage 2) | 1e-5 |
| Learning rate (stage 3) | 3e-5 |
| Weight decay (stage 1) | 1e-4 |
| Weight decay (stage 2) | 1e-6 |
| Weight decay (stage 3) | 5e-4 |
| Batch size | 32 |
| Number of epochs | 100 |
| Activation function | ReLU |
